# Environmental Particulate Matter Levels during 2017 Large Forest Fires and Megafires in the Center Region of Portugal: A Public Health Concern?

**DOI:** 10.3390/ijerph17031032

**Published:** 2020-02-06

**Authors:** Marta Oliveira, Cristina Delerue-Matos, Maria Carmo Pereira, Simone Morais

**Affiliations:** 1 REQUIMTE/LAQV, Instituto Superior de Engenharia do Instituto Politécnico do Porto, 4249-015 Porto, Portugal; cmm@isep.ipp.pt; 2 LEPABE, Departamento de Engenharia Química, Faculdade de Engenharia, Universidade do Porto, 4200-465 Porto, Portugal; mcsp@fe.up.pt

**Keywords:** forest fires, air pollution, particulate matter (PM), public health risks, WHO AirQ+ model, socio-economic impact

## Abstract

This work characterizes the dimension and the exceptionality of 2017 large- and mega-fires that occurred in the center region of Portugal through the assessment of their impact on the ambient levels of particulate matter (PM_10_ and PM_2.5_), retrieved from local monitoring stations, and the associated public health risks. PM_10_ and PM_2.5_ concentrations were increased during the occurrence of large fires and megafires, with daily concentrations exceeding the European/national guidelines in 7–14 and 1–12 days of 2017 (up to 704 µg/m^3^ for PM_10_ and 46 µg/m^3^ for PM_2.5_), respectively. PM_10_ concentrations were correlated with total burned area (0.500 < r < 0.949; *p* > 0.05) and with monthly total burned area/distance^2^ (0.500 < r < 0.667; *p* > 0.05). The forest fires of 2017 took the life of 112 citizens. A total of 474 cases of hospital admissions due to cardiovascular diseases and 3524 cases of asthma incidence symptoms per 100,000 individuals at risk were assessed due to exposure to 2017 forest fires. Real-time and in situ PM methodologies should be combined with protection action plans to reduce public health risks. Portuguese rural stations should monitor other health-relevant pollutants (e.g., carbon monoxide and volatile organic compounds) released from wildfires to allow performing more robust and comprehensive measurements that will allow a better assessment of the potential health risks for the exposed populations.

## 1. Introduction

Natural forest fires act as a catalyst for the necessary environmental change by causing the renewal of soil and air chemistry and recycling of the available nutrients. Wildfires cause replenishment of streamside vegetation and fire-adapted plants being dispersed, remove low-growing underbrush, clean the forest floor of debris, and nourish the soil. However, in the last years, some wildfires became so large and so severe that they caused strong and profound changes in the structural and functional processes of forest ecosystems and wildlife. Climate change is an undeniable reality that has been causing an increase in the frequency and intensity of extreme weather events [1,2]. Together, climate change and global warming have been strongly affecting wildland fires through the changing in weather conditions and the effects on vegetation and forest fuels [3,4,5]. Severe forest fires cause significant ecological, economic, and social problems [6,7,8,9]. Several reports indicate that forest fires frequency and severity have significantly been increasing in the European Mediterranean countries, Australia, and in North America [5,6,10,11,12,13]. Within the period 2000–2017, forest fires burned 8.5 million ha (85,000 km^2^) of European land, caused the death of 611 firefighters and civilians and an estimated economic loss of over 54 billion euros [11]. More than forty thousand fires per year occurred only in the Mediterranean countries (2010–2017) and burned 350 thousand ha (3500 km^2^) per year [11]. Some European countries, including Portugal, have been severely affected by megafires, i.e., fires that burn more than 10,000 ha (100 km^2^) of land, which are challenging the countries’ capacity to their fighting and control [11]. The World Meteorological Organization (WMO) stated that 2017 was one of the world’s three warmest years on record and presented the highest documented economic losses associated with severe weather and climate events [14]. The estimated cost of fire prevention and combat in Portugal (2000 to 2017) was around 92.5 million euros and the loss of assets and services and recovery costs were estimated in about 523 million euros, making a total cost of ca. 613 million euros [15,16]. In 2017, fires caused a direct damage in the infrastructures of 521 Portuguese companies (with a significant proportion of the industrial sector), totaling around 275 million euros, and affecting, at least temporarily, more than 4500 jobs, in 30 affected localities [15,16]. Regarding other geographical areas, Cascio [17] reported that US suppression costs with forest fires have quadrupled since 1980 indicating the higher intensity and severity of fires, and the expansion of forest-urban interface as the major reasons for the increased costs. Moreover, Rafael et al. [18] estimated that an increase of 44% in the number of fires caused an economic loss of almost 14 million dollars in Puerto Rico. However, there are costs associated with the occurrence of forest fires that are very difficult to estimate, including the costs with premature deaths and aggravation of cardio-respiratory diseases, the lost in productivity and the environmental impact in the affected areas.

**Figure 1:**
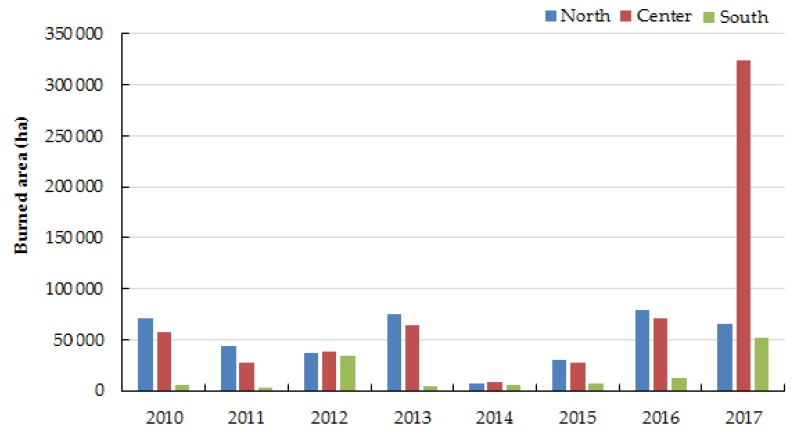
Levels of total burned area (ha) in the northern, central, and southern regions of Portugal between 2010–2017.

Regarding Portugal, 1,158,175 ha (11,581.75 km^2^) of land was burned between 2010 and 2017, representing a cumulative loss of 37% of Portuguese forest area (Table S1 of the Supplementary Material). Since 2010, the northern and central regions of the country have continuously been the most affected areas by forest fires with total annual burned area ranging from 15%–59% and from 35%–73%, respectively; the total burned area in the south region varied between 3%–31% (Figure 1). Previously, other authors covering different temporal periods reported a similar tendency in the regional distribution of forest fires in Portugal [19,20,21]. Forest fires that occurred in Portugal during the 2017 hot season devastated the center region of Portugal [5,22]. To the knowledge of these authors, no data exists regarding the environmental impact of 2017 forest fires in Portugal. The forest fire that occurred in a Spanish natural park in June 2017 caused a very negative impact on many animal and plant species including the protected ones [6]. Air contamination from the fire plume is one of the first imminent impact of forest fires that can travel thousands of kilometers downwind [23,24,25,26,27]. de la Barrera et al. [28] reported significant impact of 2017 megafires on the air quality in close and distant cities situated at more than 100 km from the burned areas. Some works were able to prove that fires may change the composition of the local water beds including rivers, sediments, and groundwater and may have a direct impact in the quality of drinking water [7]. Regarding soil, it has been reported that forest fires are able to alter soil composition and speed up its erosion because vegetation may be partially or completely destroyed and the fertile layers can even disappear, making the soil loose and easily washable by the first rains [8,29]. Moreover, the replacement of vegetation is naturally slower because ashes that cover the burned lands will promote soil alkalinity and microorganisms useful to plants did not resist to the high temperatures of fires [8].

Forest fires release large amounts of numerous hazardous pollutants to the atmosphere, including particulate matter (PM), carbon monoxide, several volatile organic compounds, and heavy metals, which represent serious risks for the health of exposed fire combat forces and populations [17,30,31,32,33]. Johnston et al. [34] estimated an interquartile range of 260–600 thousand deaths (average of 339 thousand) attributed to landscape fire smoke exposure, which included wild and prescribed fires but also agricultural, deforestation, peat, and grass fires. PM, one of the pollutants present in the fire chemical mixture, consists of a complex mixture of aerosols suspended in the air that is classified according to its aerodynamic diameter into coarse (PM ≥ 2.5 µm), fine (PM ≤ 2.5 µm), and ultrafine particles (PM < 0.1 µm) [35,36]. PM is known as human carcinogen and it has been recognized as the best indicator to estimate the health effects of ambient air pollution [35,37,38,39]. The World Health Organization (WHO) stated that PM is responsible for 3% of cardiopulmonary and 5% of lung cancer world deaths [40]. Exposure to the finest fraction of PM has been associated with an increase of 20% in human deaths and with profound negative effects on human health and ecosystems [41,42,43,44,45]. Fire emissions can account for 20% of the total daily PM_2.5_ emissions in North America during fire season [46]. Several works found positive and direct association between exposure to PM released from forest fires and all-cause mortality and respiratory morbidity including bronchitis, asthma, chronic obstructive pulmonary disease, and pneumonia [17,23,24,33,34,47]. PM_2.5_ can easily penetrate the human lungs and cause oxidative stress and inflammatory response in the cardio-respiratory system of exposed individuals, principally in the most susceptible groups of the population such as children, pregnant women, people with cardio-respiratory diseases, and the elderly [35,40,42,43,48,49,50,51,52].

Several authors have been assessing the socio-economic and environmental impact of wildfires with some works including the determination of environmental hazardous pollutants, namely PM, nitrogen and sulfur oxides, ozone, and volatile organic compounds [6,28,53,54,55,56,57,58,59,60,61]. Regarding Portugal, some studies have been reported in the last decade, having some of them estimating the emission factors of hazardous pollutants produced during large forest fires [53,54,55,58,59]. To our best knowledge, no study was found regarding the environmental monitoring of PM before and during the occurrence of large forest fire episodes in Portugal. Therefore, the main objectives of this work were to: (i) characterize the dimension and the exceptionality of 2017 forest fires in the center region of Portugal; (ii) identify the most intense periods of exposure and estimate the contribution from 2017 forest fires to environmental PM levels; (iii) evaluate the potential public health risks for the exposed populations due to the occurrence of 2017 fires.

## 2. Materials and Methods

### 2.1. Selection of Sampling Sites and Data Collection

Environmental rural monitoring stations are situated in geographical areas that are not under the direct influence of industrial sources and/or traffic routes or any other sources of anthropogenic pollution, which make them a good tool to assess the impact of forest fires. Figure S1 of the Supplementary Material presents the fourteen rural monitoring stations with background influence, under the responsibility of Portuguese Environment Agency (APA) available in Portugal. Daily mean levels of PM_10_ and PM_2.5_ were retrieved from five rural monitoring stations with background influence situated in the center region of Portugal (Coimbra, Leiria, Viseu, Castelo Branco, and Santarém; Figure S1). The district of Guarda, the third most affected locality in the center region, was not included in the analysis because it is not covered by the Portuguese environmental monitoring system (Figure S1).

Daily concentrations provided (per request) by the Portuguese Center Regional Coordination and Development Commission (CRCDC) were calculated as the mean of the 24-hourly levels; missing data, i.e., when for some reason levels were not registered at the monitoring station, were not included in the determinations. All the retrieved information was previously verified and validated by the CRCDC. Data are also available to the general public at the on-line monitoring network platform QualAr (https://qualar.apambiente.pt).

### 2.2. Data Analysis

PM_10_ and PM_2.5_ levels were expressed as median (percentile 25–75%) and range since normal distribution was not observed. Concentrations were log-transformed to increase clarity of the graphical data. Data treatment was performed in SPSS (IBM SPSS Statistics 20) and Statistica software (version 7, StatSoft Inc., Tulsa, OK, USA). Statistical significance was defined at *p* ≤ 0.05. Spearman coefficients (r) were determined to evaluate the relation between environmental levels of PM_10_ or PM_2.5_ and the total burned area.

### 2.3. Public Health Risk Assessment

Assessment of public potential health risks due to the exposure to PM_10_ and PM_2.5_ during the occurrence of 2017 large forest fires and megafires in the center region of Portugal were estimated by the World Health Organization AIRQ+ software [62,63]. Calculations performed with AIRQ+ software are based on methodologies, systematic review of meta-analysis of all the information available until 2013 and concentrations-response functions well established by different epidemiological studies [62]. Daily 24-h median concentrations of PM_10_ and PM_2.5_ in the most severe months, June and October, were used as environmental data. Relative risks allow estimating the magnitude of an association between exposure to PM and a potential disease since they indicate the likelihood of developing the potential health risk in the exposed group comparatively with the non-exposed group.

The impact of short-term exposure to PM_10_ was evaluated through the incidence of asthma symptoms in asthmatic children. The number of hospital admissions due to cardio-vascular diseases including stroke and the number of all (natural) causes of mortality in adults were estimated as short-term health risks in population exposed to PM_2.5_. Data retrieved from Pordata, a certified basis of Portuguese and European data (https://www.pordata.pt/en/Home), were used as source of the social-demographic information of the resident population in the most affected districts as well as for the health end-points baseline incidences. Table S2 (Supplementary Material) presents the information that was introduced in the AIRQ+ model application.

## 3. Results and Discussion

### 3.1. Dimension and Exceptionality of 2017 Forest Fires

**Figure 2:**
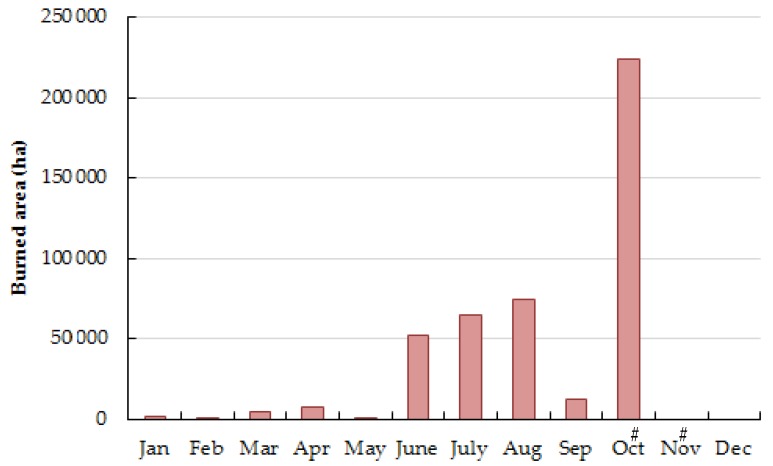
Levels of monthly total forest area (ha) burned in Portugal during 2017. # Data were not available.

In 2017, a total of 21,006 rural fire occurrences burned 539,921 ha (5399.21 km^2^) of wooded, shrub, and agricultural land, representing five times the average of the previous decennium [5,22]. During the first semester (January–June), only 13% of total annual burned area was consumed, while in the summer season and early autumn (July–October), the number of fire occurrences almost doubled and more 466,971 ha (4669.71 km^2^) of forest were burned [5,22]; Figure 2).

A total of 214 large fires, i.e., fires that burned more than 100 ha (1 km^2^), were registered in Portugal with 107 occurring in the center region and burning 79% of annual total burned area (26% in Coimbra, 14% in Guarda, 12% in Castelo Branco, 10% in Viseu, 9% in Leiria and 8% in Santarém [5,22]). A total of nine megafires were registered and burned more than 230 thousand ha (2300 km^2^) of land, being 67% of those fires initiated in October, 22% in June and 11% in July (Figure 2) [5,22]. Moreover, June and October were the most severe months at the center region of the country with the occurrence of 13 large- and eight mega-fires in short periods of time, which affected public health and caused important socio-economic, and environmental damages [64]. The accumulation of biomass in uncultivated lands and abandoned forests strongly contributed to the dimension of the registered wildfires. In addition, around 50% of the investigated cases of rural fires were attributed to human negligent behavior, mainly through the incorrect use of fire, while intentional acts (incendiarism) represented 32% of the identified fire causes [64].

### 3.2. Levels of PM_10_ and PM_2.5_ during 2017

Overall, PM_10_ annual median concentrations ranged from 8.5 µg/m^3^ at the district of Viseu to 17.5 µg/m^3^ at Coimbra (Table S3 of the Supplementary Material). Regarding PM_2.5_, levels varied between 4 (Castelo Branco) to 7 µg/m^3^ (Santarém); data from the district of Leiria were insufficient (Table S3).

**Figure 3:**
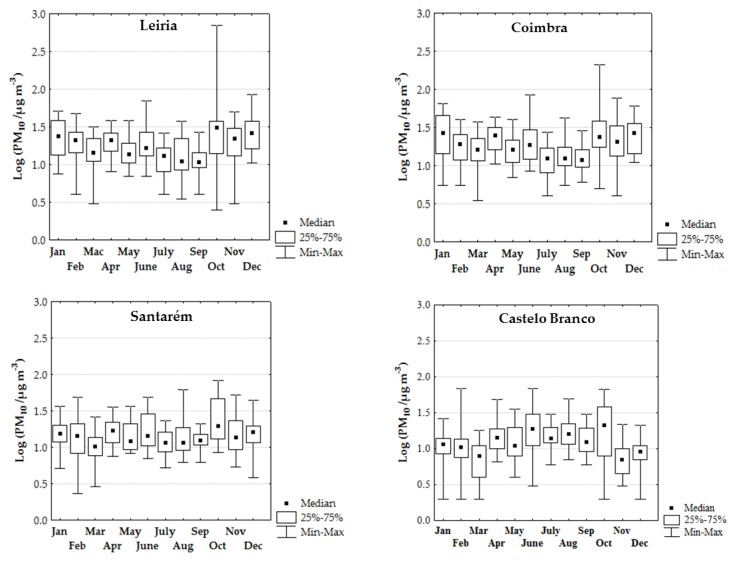

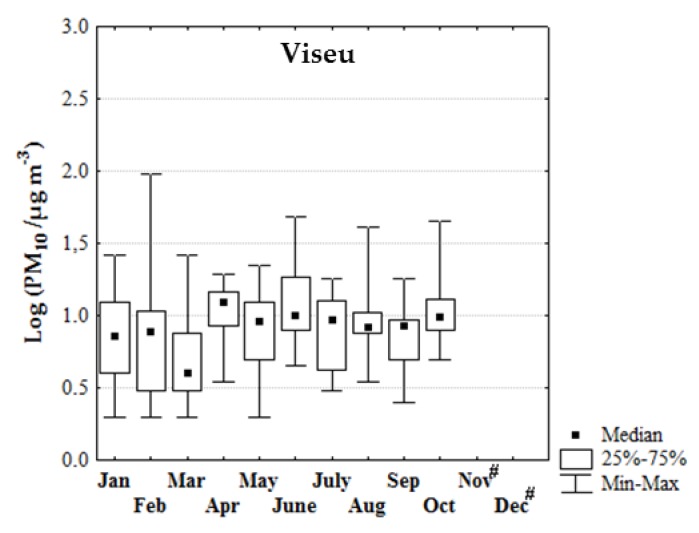
Log-transformed monthly concentrations of particle matter, PM_10_ (median, percentiles 25–75, and range; µg/m^3^) in the ambient air of the center region of Portugal. # Data were not available; * Data were only available until the 10 October.

**Figure 4:**
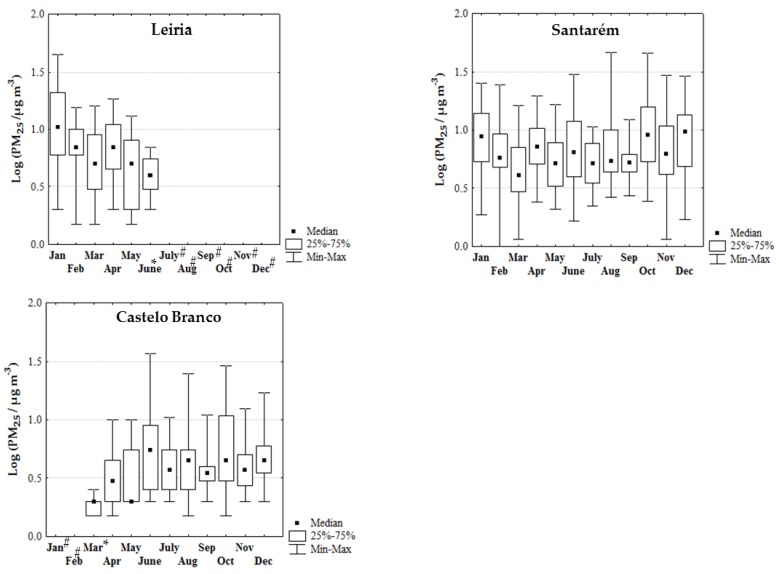
Log-transformed monthly concentrations of PM_2.5_ (median, percentiles 25–75, and range; µg/m^3^) in the rural monitoring stations from the districts in the center region of Portugal. # Data were not available; * data were only available until the 16 June; ** data were only available after the 21 March.

Figure 3 presents the log-transformed monthly median concentrations of PM_10_ in the ambient air of the center districts of Portugal between January to December 2017. PM_10_ inter-district comparison followed the sequence: Leiria (11–31 µg/m^3^) > Coimbra (12–27 µg/m^3^) > Santarém (10–20 µg/m^3^) ≈ Castelo Branco (7–21 µg/m^3^) > Viseu (4–13 µg/m^3^). Global range of PM_10_ levels were predominantly higher in the summer (June to August) and early autumn (October) than in the winter (December and January–March) season for each district considered (11–31 µg/m^3^ versus 15–26 µg/m^3^ at Leiria; 11–20 µg/m^3^ versus 10–16 µg/m^3^ at Santarém; 12–21 µg/m^3^ versus 8–11 µg/m^3^ at Castelo Branco; 8–10 µg/m^3^ versus 4–8 µg/m^3^ at Viseu), except at Coimbra (12–24 µg/m^3^ versus 16–27 µg/m^3^; Figure 3). Regarding PM_2.5_, a similar inter-district comparison was found: Leiria (3–10 µg/m^3^) > Santarém (4–9 µg/m^3^) > Castelo Branco (2–5 µg/m^3^); data were not available in the districts of Coimbra and Viseu (Figure 4). Global range of PM_2.5_ monthly median concentrations were similar between the hottest months and the winter period (5–9 µg/m^3^ versus 4–9 µg/m^3^ at Santarém and 4–5 µg/m^3^ versus 2–5 µg/m^3^ at Castelo Branco) (Figure 4). No comparison between seasons could be done for the district of Leiria because data were only available until June 2017.

PM_10_ and PM_2.5_ monthly median concentrations were moderately to strongly correlated in the districts of Castelo Branco (r = 0.488; *p* = 0.153), Santarém (r = 0.854; *p* ≤ 0.001) and Leiria (r = 0.941; *p* = 0.005), thus revealing the predominance of common sources. Concerning correlations between these pollutants and monthly total burned area, moderate to high correlations were attained, namely r = 0.500 for Leiria, r = 0.696 for Viseu and r = 0.949 for Coimbra (*p* > 0.05). Moreover, positive and moderate correlations were also found between monthly PM_10_ concentrations with monthly total burned area/distance^2^ for the districts of Coimbra, Viseu, and Santarém (0.500 < r < 0.667; *p* > 0.05); PM_10_ levels were statistically insufficient for the district of Leiria while PM_2.5_ data were insufficient to assess this correlation for all the districts. de la Barrera et al. research [28] also found significant positive correlations between the ambient concentrations of PM_10_ and PM_2.5_ with the burned area/distance^2^ in South–Central Chilean cities due to the occurrence of megafires in 2017 summer. Dry conditions with high temperatures and rainfall below 10–25 mm were the most widespread meteorological conditions through the Portuguese territory in 2017, principally in the months of April, and between June to October [65]. Comparatively with the period 1971–2000, summer of 2017 presented deviations of mean air temperature that varied between −0.5 (July and August) to 6.5 °C (October) [65]. As observed in the center region of Portugal during 2017, Canadian wildfires were preceded by long periods of drought and strong winds on July 2002 [23].

### 3.3. Levels of PM during the Occurrence of 2017 Large Fires and Megafires

#### 3.3.1. June

On 17 June, two megafires and three large forest fires started at the center region (Leiria, Coimbra, Castelo Branco, and Santarém). During this day, there was a weak to moderate wind from the northwest and it increased in speed until to 6 p.m., then turned to the east quadrant and hardened, which was caused by a gust front coming from the convective cells (thunderstorm) approaching the region [15]. These megafires started with a difference of nine minutes and were caused by electric discharges mediated by local energy distribution network in the presence of a dry thunderstorm [15]. On 18 June, the meteorological conditions were still particularly severe, which contributed to the severity of fires which were extinguished only on 24 June. Overall, more than 45 thousand ha (450 km^2^; 10% of annual total burned area) were burned between days 17 to 24 [22]. The two megafires at Leiria and Coimbra were respectively the second and eighth largest ever in Portugal and they affected eleven counties from the center region.

**Figure 5:**
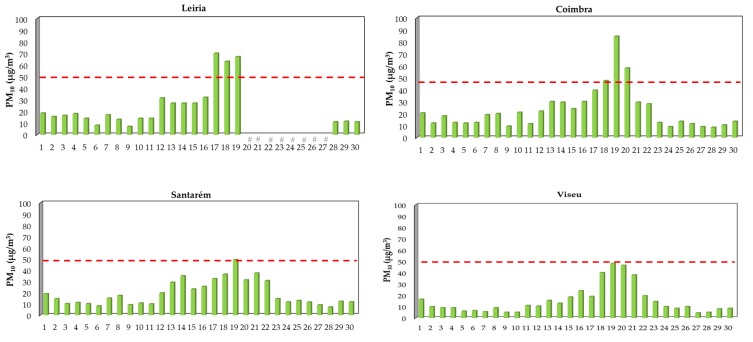

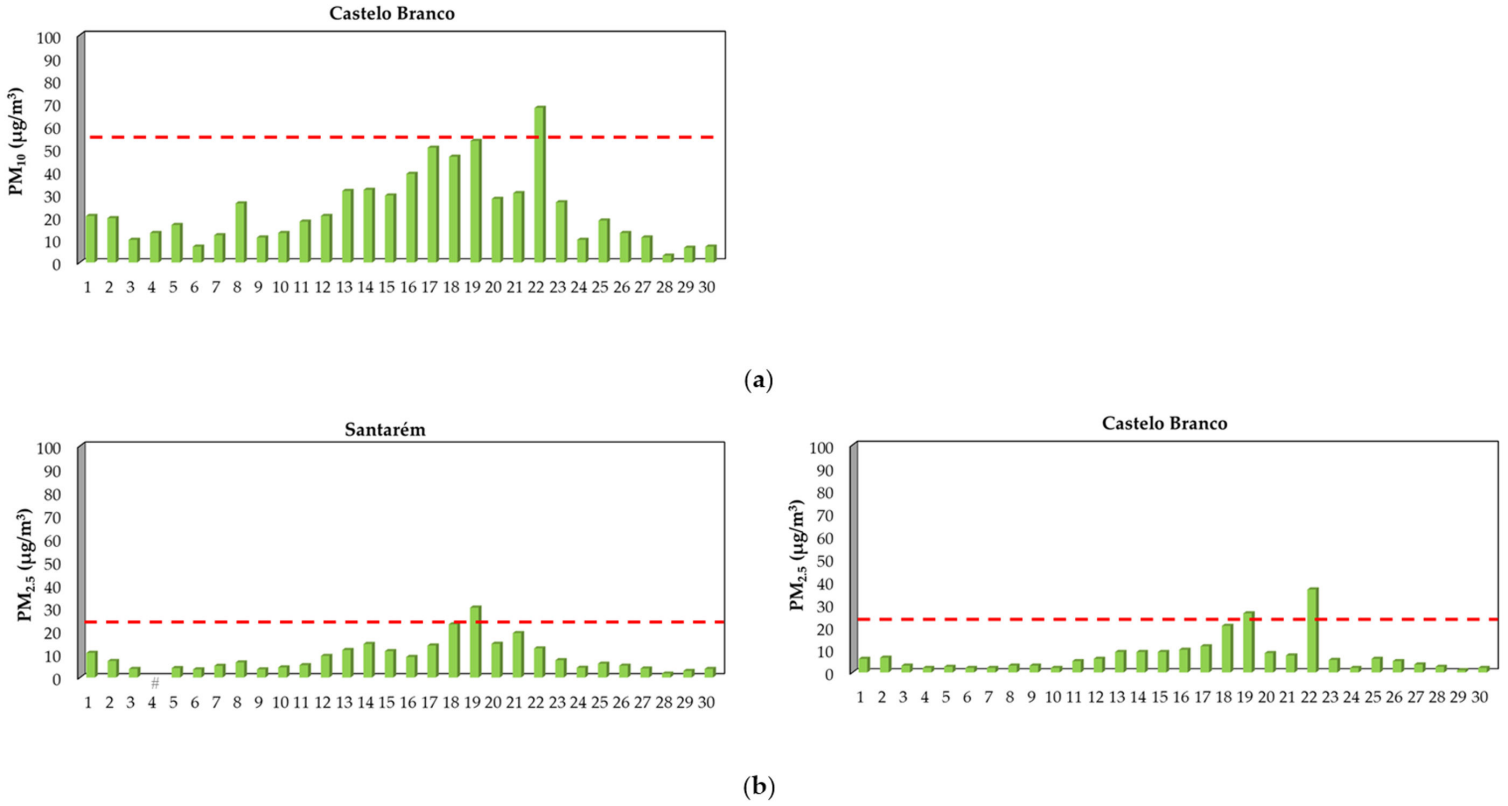
Daily concentrations (median, µg/m^3^) of PM_10_ (**a**) and PM_2.5_ (**b**) in the rural monitoring stations from the center region of Portugal during the month of June. Dashed lines represent the WHO guidelines of 50 and 25 µg/m^3^ [36]. # Data were not available.

Figure 5 presents the daily concentrations of PM_10_ and PM_2.5_ in the center region of Portugal during the month of June. Overall, PM_10_ levels varied between 10 (Viseu) to 19 µg/m^3^ (Castelo Branco), being the concentrations increased during the occurrence of the large forest fires; increments started on day 17 and reached daily maximum levels that ranged from 48 (day 19 at Viseu) to 85 µg/m^3^ (day 19 at Coimbra) (Figure 5a). Regarding PM_2.5_ concentrations, a similar profile was observed with daily maximum levels reaching 30 (day 19) and 37 µg/m^3^ (day 22), respectively at the districts of Santarém and Castelo Branco (Figure 5b). In addition, on June 2017, a megafire started at the southeast of Spain and consumed more than 10 thousand ha (100 km^2^) of land in approximately 60 h [6]. Ambient concentrations of hazardous pollutants released during this large fire were extremely high with maximum values of 1000 µg/m^3^ for PM_10_, 100 µg/m^3^ for carbon monoxide, 951 µg/m^3^ for ozone, 478 µg/m^3^ for nitrogen dioxide, and 116 µg/m^3^ for sulfur dioxide [6]. However, the levels registered for carbon monoxide, nitrogen dioxide and PM_10_ reached the upper limits of the instruments’ operation and thus concentrations observed during the occurrence of that fire could have been even higher [6]. Plume smoke from Spanish fires affected large metropolitan areas near the entire Mediterranean coast and 48 to 96 h after fire suppression were needed to recover to the initial atmospheric levels [6].

#### 3.3.2. October

On 15 October, thirty-eight large fires started in the national territory with six megafires and 10 large fires situated at the center region, which represented 94% of total burned area [22]. The main causes of these fires were incendiarism (36%), biomass burnings (33%), and re-ignitions (24%) [16]. Initially, fires were strongly influenced by Hurricane Ophelia since it was the force of the wind and the low humidity that allowed their growth [14,16]. However, the occurrence of pyro-convective phenomena strongly contributed to the great episode of fires. This specific sequence of events constitutes the largest pyro-convective phenomenon so far registered in Europe and the world’s largest in 2017, with an average of 10 thousand ha (100 km^2^) burned per hour during approximately thirteen consecutive hours [16].

**Figure 6:**
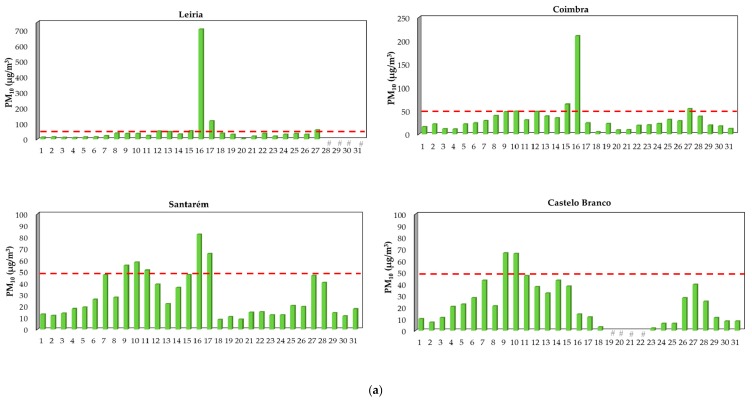

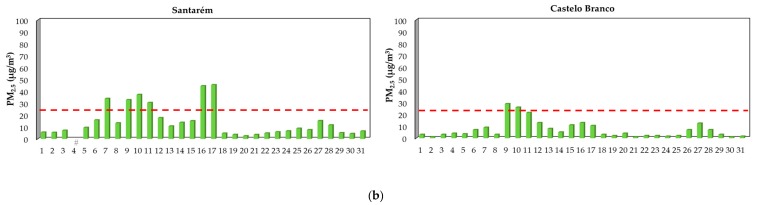
Daily concentrations (median, µg/m^3^) of PM_10_ (**a**) and PM_2.5_ (**b**) in the rural monitoring stations from the center region of Portugal during the month of October. Dashed lines represent the WHO guidelines of 50 and 25 µg/m^3^ [36]. # Data were not available.

Daily PM_10_ median concentrations ranged between 20 (Santarém) to 31 µg/m^3^ (Leiria) with levels being increased between days 15 and 18 (Figure 6a). The monitoring station from the district of Viseu only recorded information until day 10 and thus PM data during the occurrence of October’ fires was not registered (Figure 3). PM_10_ levels reached daily maximum values that varied between 67 (day nine at Castelo Branco) to 704 µg/m^3^ (day 16 at Leiria). On day 16, one day after starting fires, extremely high concentrations of PM_10_ were registered in the district of Leiria, with hourly levels of 1000 µg/m^3^ over five consecutive hours; afterwards concentrations started slowly to decrease and reached pre-fire values after 52 h (Figure S2 of the Supplementary Material). It is important to mention that during those five consecutive hours, the PM_10_ sensor reached its maximum detectable concentrations, and therefore the observed concentrations might have been higher.

Regarding PM_2.5_, daily median levels ranged between 4 (Castelo Branco) to 9 µg/m^3^ (Santarém), with maximum values reaching 46 µg/m^3^ at Santarém (Figure 6b). Two studies monitored the levels of PM_2.5_ during the occurrence of Canadian forest fires: one reported daily concentrations that reached 86 µg/m^3^, a 30-fold increase in the indoor air of asthmatic children’s bedroom [66] and the other one found maximum ambient concentrations of 338 µg/m^3^ during the peak period of haze over the affected cities [67]. More recently, McClure and Jaffe [57] concluded that concentrations of ambient PM_2.5_ is increasing in the northwest of USA because of the increase in the intensity and severity of wildfires. Unfortunately, data about other air pollutants (e.g., ozone, carbon monoxide, volatile organic compounds, nitrogen, and sulfur dioxides) monitored during 2017 forest fires in the center region of Portugal were not measured in the five characterized rural stations since only some of those pollutants are included in the Portuguese monitoring program. Some data were available for ozone, nitrogen and sulfur dioxides, however they were insufficient to evaluate the impact of forest fires emissions.

#### 3.3.3. PM Exceedances in the Center Region

During 2017 (January–December), a total of seven (Castelo Branco) to fourteen (Coimbra) days presented daily PM_10_ median levels that exceeded the national and international guidelines of 50 µg/m^3^, being 36–88% of the exceedances registered in the hot season principally in October [68,69]. The highest PM_10_ levels were registered in the months of October and June during the periods when large fires and megafires occurred; concentrations were up to 2 (82.3 µg/m^3^ at Santarém), 4 (210 µg/m^3^ at Coimbra), and 14 (704 µg/m^3^ at Leiria) times higher than the defined guideline of 50 µg/m^3^ (Figure 5a and Figure 6a). Previously, Liu et al. [47] performed a systematic review with all the data published since 1986 to May 2014 regarding wildfire emissions and concluded that PM_10_ levels were 1.2 to 10 times higher during the occurrence of forest fires. However, the number of PM_10_ exceedances in the center region of Portugal was below the “no more than 35 days over a calendar year” recommended by WHO [36]. Regarding PM_2.5_, daily levels exceeded the guideline of 25 µg/m^3^ in 1 (Castelo Branco) to 12 (Santarém) days, with up to 83% of exceedances being observed in the months of June, August, and October (Figure 5b and Figure 6b). PM_2.5_ exceedances may be under-estimated since the monitoring station of Leiria only registered information until June 16th (Figure 4). Since data about environmental exposure to ambient PM_10_ (and PM_2.5_) are inexistent for the district of Guarda, one of the most affected districts by 2017 fires, it is expected that PM_10_ (and PM_2.5_) exceedances would be higher.

It is worth to mention that PM_10_ and PM_2.5_ exceedances were also observed in some days of the cold season, namely on November (Leiria, Coimbra, and Santarém), December (Leiria, Santarém, and Coimbra), January (Leiria, Santarém, and Coimbra), and February (Viseu and Castelo Branco). It is a regular practice to use biomass burning to clean lands and forests, especially during the cold months since in the fire season it is prohibited in Portugal.

### 3.4. Public Health Impact of Fires

In 2017, forest fires caused 112 human deaths and important social, economic, and environmental losses [14,15,16]. Forest fires affected not only private forest areas, but also agricultural areas, national forests, business infrastructures, equipment municipalities, rural tourism facilities and private dwellings. The worst single megafire incident took place in June at the district of Leiria (Pedrogão Grande) and took the lives of 64 people [15]. These fatalities were concentrated in an area of 20 km^2^ [16]. The destruction of patrimony and patrimonial assets caused the partial and/or complete destruction of about 490 houses and nearly half a hundred industrial units in various sectors with significant losses in equipment and infrastructures [15]. Concerning the wildfires of October, they took the lives of 48 citizens [16]. About 85% of the victims were permanent residents in their respective areas, which proves the large geographic dispersion of these fires since victims lived in thirty different localities over an area of about 4000 km^2^ [16].

WHO AIRQ+ software was used to estimate the magnitude of the impacts of ambient air pollution during the months of June and October on the health of exposed populations from the center region of Portugal. The estimated attributable fraction represents the fraction of the health outcome in a specific population that can be attributed to the exposure to PM_10_ and PM_2.5_ in a given population assuming the causal association between exposure to PM and the considered health outcome and that no confounding effects on this association were present [62,63]. The number of estimated attributable proportions and the attributable number of cases among the population at risk are exhibited in Table 1. Impact of short-term exposure to PM_10_ was evaluated through the incidence of asthma symptoms in asthmatic children. Overall, it was estimated the occurrence of 958 and 2566 cases per 100,000 population at risk in the center region of Portugal during the months of June and October, respectively. In these months, Leiria and Coimbra districts presented the highest number of attributable cases while Viseu exhibited the lowest ones (307 and 276 versus 99 in June; 582 and 1455 versus 105 in October; Table 1). However, evaluation of short-term exposure to PM_10_ during live forest fire events may be underestimated because one of the most affected regions, Guarda, is not included in the national environmental monitoring system (Figure S1). Moreover, the number of cases determined for October was 1 (Viseu and Castelo Branco) to 5 (Leiria) times higher than during June, which was directly associated with the increased number of daily PM_10_ concentrations higher than the available guideline (50 µg/m^3^; [36,68,69]. Again, the number of estimated cases of short-term effects due to the exposure to PM_2.5_ was predominantly higher in October comparatively with June. The attributable proportion of hospital admissions due to cardiovascular diseases including stroke varied between 0.17% at Castelo Branco to 0.60% at Santarém and a total of 244 and 474 cases were estimated in June and October, respectively (Table 1); no PM_2.5_ levels were available for other districts (Table S2). In October 2017, the number of hospital admissions due to cardiovascular diseases was double for people living at Santarém comparatively with those living at Castelo Branco (332 versus 142 cases per 100 000 population at risk; Table 1). A total of 21 deaths (7 in Santarém and 14 in Castelo Branco) were estimated by AIRQ+ software due to the environmental exposure to PM_2.5_ during the occurrence of large forest fires (Table 1). A total of 14 deaths were estimated by the AIRQ+ for the month of October which represented 29% of the real deaths registered in Portugal for that month. The number of estimated cases would be expected to increase if the levels of PM_2.5_ were available for the other districts, principally Coimbra, Leiria, and Guarda, which were the most affected districts by 2017 forest fires (Table S1). The potential health risks estimated by AIRQ+ carry some uncertainties because the model was constructed with data collected from different studies mainly performed in Western European countries and North America. Relative risks assumed by the software represent average values that may differ across and between different areas. Moreover, the AIRQ+ model assumed that environmental levels of PM_10_ and PM_2.5_ affect equally all the exposed population regardless of their age and/or health condition and it does not account for multiple exposure routes and multipollutant scenarios. Despite these limitations, the AIRQ+ model have been used to estimate the public potential health risks of exposure to PM [44,70]. Previously, Analitis et al. [71] proved that exposure to forest fires significantly increased the daily total number of cardiovascular (6% for medium fires versus 61% for large fires) and respiratory (16% and 92% for medium and large fires, respectively) deaths, being cardiovascular effects more pronounced in younger people while respiratory effects were predominant in the older population. Reid et al. [33] found strong associations between the increased risk of respiratory infections and all-cause mortality; results for cardiovascular effects were generally inconclusive. In a recent systematic review (of the studies from 1986–2014), a significant association between wildfire emissions and the increased risk of respiratory morbidity was found [47]. Reid et al. [33] also reported strong associations between exposure to forest fire emissions and the exacerbation of asthma and chronic obstructive pulmonary disease. American elderly people presented increased rates of hospitalizations due to cardiovascular and respiratory diseases after the exposure to the smoke plume of Canadian wildfires (PM_2.5_ levels of 53 µg/m^3^ [23]). Moreover, Viswanathan et al. [24] reported significant increase in hospital emergency room visits for eye irritation, smoke inhalation, the aggravation of asthma and other respiratory pathologies due to the exposure to high levels of PM after a large wildfire that occurred in San Diego, USA. Positive and strong associations between exposure to PM released from fires and cardio-respiratory diseases have been reported, being children, pregnant women, elderly, people with chronic diseases, and firefighters the most vulnerable groups [17,47,72].

## 4. Conclusions

The present work highlights the public health implications that occurrence of forest fires can cause in exposed populations and in environment. Since limited data were available for the ambient levels of PM_2.5_, it is recommended the inclusion of this pollutant in all the Portuguese rural monitoring stations. Moreover, data retrieved from rural monitoring stations with background influence may not represent the levels of exposure to PM_10_ and/or PM_2.5_ of the entire population living in that district. Still, it is believed that this is an approximation of the real levels of exposure experienced by those populations during the occurrence of large forest fires.

A total of 112 deaths (64 people in June and 48 people in October) were registered in Portugal as well as important social, economic, and environmental losses as a direct consequence of 2017 forest fires. WHO AIRQ+ software estimated a total of 21 deaths (29% of the registered deaths), more than 450 cases of hospital admissions due to cardiovascular diseases and 3500 cases of asthma incidence symptoms per 100,000 individuals at risk. These health risks were attributed to PM_10_ emissions during the occurrence of large forest fires. Future studies should include the monitoring of other health-relevant air pollutants (e.g., carbon monoxide and volatile organic compounds) to better characterize the impact of forest fire emissions in the local populations.

At this point, there is a clear need to develop programs of awareness, and self-protection of populations, principally in the most susceptible groups including children, pregnant women, people with chronic diseases, and elderly, based on principles of subsidiarity and sustainability of interventions. Simulations of the early development of fire behavior indicate that most megafires would not be controllable beyond three to five minutes (terrestrial resources) or 10–17 minutes (aerial resources) after ignition. On October 2017, most of the occurrences were fought within five to ten minutes after the alert, but the first intervention occurred when the fire was already beyond the extinction capacity—the reason why the fire combat options were much reduced against the extreme behavior of the fire. Megafires will continue to happen in the near future through the combination of unfavorable meteorological conditions because of climate change and global warming, and thus, important actions need to be implemented in order to protect human lives and the impact on the ecosystems. Some of these actions should be related with raising awareness of the population towards greater citizenship and adopting a territorial culture that guarantees personal defense against catastrophes and defines local means to face them. An adequate and on-time smoke, fuel, and forest land management system also assumes a crucial key role in fire behavior, prevention, and in the protection of population health. Designing a robust information system that allows the population to be broadly covered and that effectively disseminates alerts and warnings at critical times would also help to protect human lives. Local strategies (e.g., use of controlled fires) to reduce the biomass build-up should be implemented to prevent the accumulation of potential forest fuels during the hot season. Methodologies able to monitor PM levels on real-time and in situ should be combined with action plans to better protect public health.

## Figures and Tables

**Table 1:** Estimated attributable proportion (%) and cases (per 100,000 population at risk) of short-term health end-points estimated by AIRQ+ software [63] for the regions that included the districts under study due to PM_10_ and PM_2.5_ exposure levels in June and October 2017.

Region	Health End-Point	Pollutant	Month	Relative Risk	Attributable Proportion	Attributable Cases
				Median (95% Confidence Interval)		
Coimbra *	Incidence of asthma symptoms in asthmatic children	PM10	June	1.03 (1.01–1.05)	3.62 (0.78–6.57)	307 (66.0–558)
			October	1.03 (1.01–1.05)	6.85 (1.43–12.9)	582 (122–1093)
Leiria *	Incidence of asthma symptoms in asthmatic children	PM10	June	1.03 (1.01–1.05)	4.13 (0.89–7.50)	276 (59.3–500)
			October	1.03 (1.01–1.05)	21.8 (3.13–55.7)	1455 (209–3714)
Viseu *	Incidence of asthma symptoms in asthmatic children	PM10	June	1.03 (1.01–1.05)	1.30 (0.40–3.41)	99.0 (21.2–181)
			October	1.03 (1.01–1.05)	1.99 (0.42–3.65)	105 (22.5–193)
Castelo Branco	Incidence of asthma symptoms in asthmatic children	PM10	June	1.03 (1.01–1.05)	3.83 (0.83–6.91)	144 (31.2–261)
			October	1.03 (1.01–1.05)	4.33 (0.94–7.82)	163 (35.3–295)
	Hospital admissions: cardio–vascular diseases including stroke	PM2.5	June	1.01 (1.00–1.02)	0.17 (0.03–0.31)	138 (25.7–252)
			October	1.01 (1.00–1.02)	0.17 (0.03–0.32)	142 (26.5–258)
	Mortality, all (natural) causes (adults ≥ 30 years)	PM2.5	June	1.01 (1.00–1.02)	0.23 (0.08–0.37)	3.72 (1.36–6.10)
			October	1.01 (1.00–1.02)	0.23 (0.09–0.38)	3.82 (1.40–6.24)
Santarém	Incidence of asthma symptoms in asthmatic children	PM10	June	1.03 (1.01–1.05)	2.61 (0.56–4.71)	132 (28.4–238)
			October	1.03 (1.01–1.05)	5.17 (1.12–9.31)	261 (56.4–469)
	Hospital admissions: cardio–vascular diseases including stroke	PM2.5	June	1.01 (1.00–1.02)	0.19 (0.04–0.35)	106 (19.8–193)
			October	1.01 (1.00–1.02)	0.60 (0.11–1.09)	332 (61.9–605)
	Mortality, all (natural) causes (adults ≥ 30 years)	PM2.5	June	1.01 (1.00–1.02)	0.26 (0.09–0.42)	3.18 (1.17–5.20)
			October	1.01 (1.00–1.02)	0.31 (0.30–1.32)	9.96 (3.64–16.3)

* Data on PM_2.5_ levels were not available and PM_2.5_ short-term health end-points could not be estimated.

## Supplementary Materials

The following are available online at https://www.mdpi.com/1660-4601/17/3/1032/s1, Table S1: Annual burned area (ha) of forest in the eighteen districts of Portugal (2010–2017), Table S2: Data summary used in the World Health Organization AIRQ+ software, Figure S1: Geographical localization of the rural monitoring stations with background influence in Portugal, Figure S2: PM_10_ hourly mean concentrations on 15 to 17 October at the rural monitoring station of Leiria.
