# Supplementary material for: Environmental Particulate Matter Levels during 2017 Large Forest Fires and Megafires in the Center Region of Portugal: A Public Health Concern?
Source: Int J Environ Res Public Health. 2020 Feb 6;17(3):1032. doi: 10.3390/ijerph17031032 (PMC7036973; doi:10.3390/ijerph17031032)
Supplement: Supplementary file 1 [file ijerph-17-01032-s001.pdf]

Supplementary Material

Environmental Particulate Matter Levels During 2017 Large Forest Fires and Megafires in the Center Region of Portugal: A Public Health Concern?

Marta Oliveira [1,*], Cristina Delerue-Matos [1], Maria Carmo Pereira [2] and Simone Morais [1,*]

[1] REQUIMTE/LAQV, Instituto Superior de Engenharia do Instituto Politécnico do Porto, 4249-015 Porto, Portugal; cmm@isep.ipp.pt

[2] LEPABE, Departamento de Engenharia Química, Faculdade de Engenharia, Universidade do Porto, 4200-465 Porto, Portugal; mcsp@fe.up.pt

* Correspondence: mrta.olveira@graq.isep.ipp.pt (M.O.); sbm@isep.ipp.pt (S.M.); Tel.: +351 228340500 (S.M.)

Table S1. Annual burned area (ha) of forest in the eighteen districts of Portugal (2010-2017).

**Year**	**North**					**Center**						**South**							
	Viana Castelo	Braga	Porto	Vila Real	Bragança	Aveiro	Coimbra	Leiria	Castelo Branco	Viseu	Guarda	Santarém	Lisboa	Setúbal	Portalegre	Évora	Beja	Faro	Total
2010	24246	14395	8552	18466	4771	8299	1486	1247	1253	19331	25736	1199	824	2263	218	472	229	104	133090
2011	5606	7510	4770	14263	11137	2675	588	2123	3156	7402	11556	425	1397	211	164	490	221	119	73813
2012	2917	8931	3664	6980	14824	2691	5649	2495	2018	15445	10219	7804	1738	361	1177	341	795	22183	110232
2013	12451	12025	14087	23209	23097	3224	3184	2329	3119	42009	9818	636	904	480	788	291	577	530	152758
2014	929	766	1362	1077	2274	313	1302	84	304	1075	5306	249	281	360	2489	407	595	756	19929
2015	10031	7154	2527	5445	5264	3498	1709	1028	2817	6573	12016	2266	990	294	205	986	1182	427	64412
2016	31440	14709	13074	12350	7191	42344	2085	1198	2495	10413	11943	3888	607	372	982	282	417	5734	161523
2017*	8551	11898	7687	15770	22029	11859	113839	38800	52721	46868	60038	35570	1275	2869	10922	454	1039	249	442418

* Data retrieved from the provisional report [18].

Table S2. Data summary used in the World Health Organization AIRQ+ software.

AIRQ+ input data	Coimbra	Leiria	Viseu	Castelo Branco	Santarém
Social-demographic data [a]					
Resident population	438228	287040	255780	82273	239346
Area size (km^2)	4336	2449	3238	4615	4275
Environmental data [b]					
June - PM_{10} (mean and range; $\mu g/m^3$)	23 (9-85)	25 (7-70)	15 (5-48)	23 (3-68)	19 (7-49)
June - $PM_{2.5}$ (mean and range; $\mu g/m^3$)	n.a.	n.a.	n.a.	7.6 (1-37)	8.8 (2-30)
October - PM_{10} (mean and range; $\mu g/m^3$)	34 (18-39)	57 (3-705)	16 (5-45)	25 (2-67)	29 (9-82)
October - $PM_{2.5}$ (mean and range; $\mu g/m^3$)	n.a.	n.a.	n.a.	7.5 (1-29)	14 (2-46)
Health end-points baseline incidences [c]					
External consultations in hospitals - medical pediatrics	37215	19144	13556	3102	12066
Urgencies in hospitals	367052	177399	200487	67300	13716
Total deaths of residents in Portugal	5573	3177	3228	1346	2975

[a] Social-demographic data is presented for the entire region where each district is included; data retrieved from Pordata (https://www.pordata.pt/Subtema/Municipios/Popula%C3%A7%C3%A3o+Residente-214);

[b] Overall monthly median values determined with the data retrieved from the environmental monitoring stations (Figures 6 and 7)

[c] Health end-points baseline incidences were retrieved from Pordata (https://www.pordata.pt/Subtema/Municipios/Servi%C3%A7o+Nacional+de+Sa%C3%BAde+(SNS)-219);

n.a. – information not available.

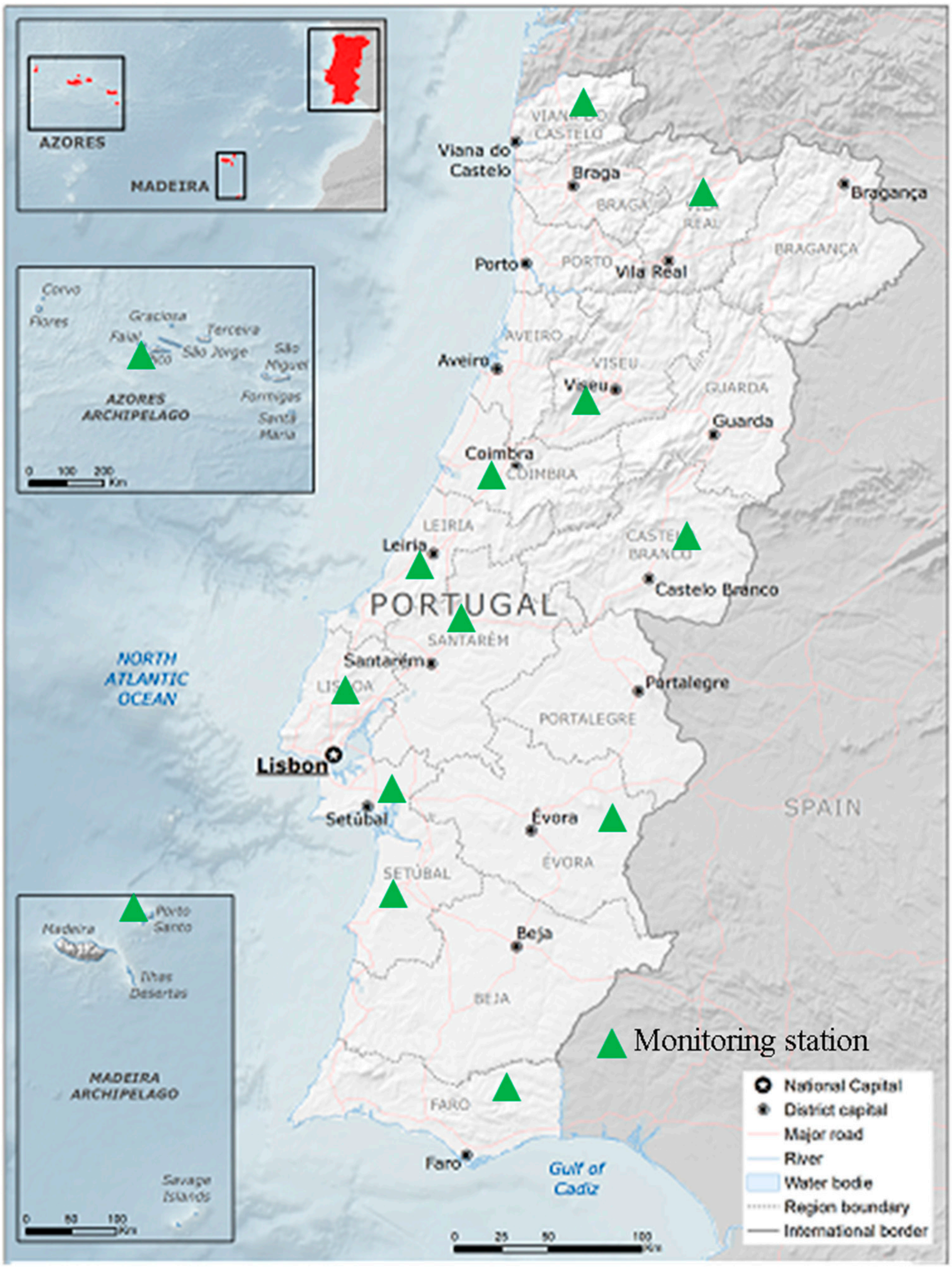

Source: https://erccportal.jrc.ec.europa.eu/Maps/Base-maps-catalogue/mapId/2282/xmps/1708.

Figure S1. Geographical localization of the rural monitoring stations with background influence in Portugal.

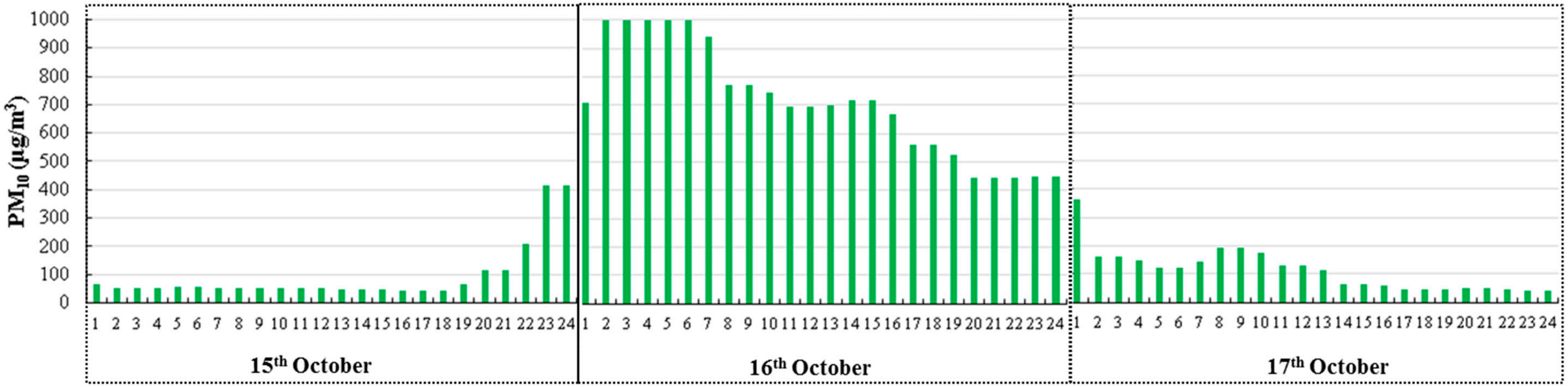

Figure S2. PM_{10} hourly mean concentrations on October 15th to 17th at the rural monitoring station of Leiria.
